# Expectations don’t protect us from emotional distractions

**DOI:** 10.3758/s13414-025-03085-8

**Published:** 2025-05-23

**Authors:** André Botes, Imogen A. Moore, Gina M. Grimshaw

**Affiliations:** 1 School of Psychology, Te Herenga Waka, Victoria University of Wellington, PO Box 600, Wellington, 6012 New Zealand; 2 Te Pūnaha Matatini, The New Zealand Centre for Research Excellence in Complexity, Auckland, New Zealand; 3 School of Psychology, Waipapa Taumata Rau, University of Auckland, Auckland, New Zealand

**Keywords:** Attention, Emotion, Distraction, Reactive control, Proactive control, Dual mechanisms of control, Emotional distraction

## Abstract

We often attend to irrelevant information to the detriment of our goals. Emotional stimuli, in particular, capture attention effectively. Usually, this capture is adaptive – alerting us to possible threats or rewards – but can be costly when attention is required elsewhere. Previous studies show that we are less distracted by emotional stimuli when they appear frequently, consistent with the claim that expectation of upcoming conflict encourages the use of effective proactive attentional control. An alternative explanation, however, is that better attentional control arises through greater experience with frequent distractors. To distinguish between these alternatives, we conducted three experiments that tested the effects of expectation on attentional control of emotional distractors while holding experience constant. Participants performed a simple letter identification task while emotionally neutral or negative task-irrelevant images also appeared on 25% of trials, either predictably (on every fourth trial) or unpredictably. As expected, emotional images were more distracting than neutral ones. However, predictability of upcoming emotional distractors did not improve participants’ ability to ignore them; indeed, it sometimes made distraction significantly worse. Similar findings were observed even when participants received incentives to use the sequential presentation of distractors to improve performance. Our findings imply that simply expecting distraction to occur does not help to prevent it.

**Supplementary Information:**

The online version contains supplementary material available at 10.3758/s13414-025-03085-8.

## Introduction

The limited nature of attention resources demands mechanisms that control where and when we direct our attention, ensuring that we process what is relevant to our goals while not being distracted by the many things which are not. Preventing distraction, however, becomes more difficult when distractors are more salient. Emotional stimuli especially out-compete emotionally neutral, less salient stimuli for limited attentional resources (Carretié, 2014; Pool et al., 2016). Emotional distractors may therefore place exceptional demands on cognitive and attentional control mechanisms that serve to help us achieve goals.

The Dual Mechanisms of Control framework (the DMC framework; Braver, 2012; Braver et al., 2009) posits that we enact cognitive control through either proactive or reactive mechanisms triggered by response conflict (Botvinick et al., 2001) or perceptual/attentional conflict (Chelazzi et al., 2019; Di Bello et al., 2022; Geng, 2014). Within the framework, proactive control mechanisms involve the maintenance of goal relevant information, allowing people to bias attention/resources optimally, *prior* to conflict occurring. They are therefore preventative. Although very effective, these mechanisms are computationally costly and are used only when the benefits of control offset the cost of their implementation. In contrast, less costly reactive control mechanisms are corrective, used only after conflict has been detected, to bring attention or behaviour back in line with our goals. Reactive control mechanisms are therefore relied on as a ‘default state’, which allows for less costly but less effective control. The key to efficient control then is to ‘meter-out’ the relative use of proactive and reactive control, given contextual needs, benefits, and costs.

Proactive control should be advantageous when added benefit is provided for effective cognitive control, such as when reward is provided for good performance (Locke & Braver, 2008; Padmala et al., 2017; Walsh et al., 2018, 2021), or when the costs invested in proactive mechanisms are more likely to bear fruit, such as when upcoming distractors can be expected (Braver, 2012; Braver et al., 2009). Expectation has previously been manipulated by increasing the frequency with which conflict-inducing events occur (Burgess & Braver, 2010; Won et al., 2019). In emotional contexts, studies employing task-irrelevant emotional distractor paradigms (Augst et al., 2014; Grimshaw et al., 2018; Micucci et al., 2020; Murphy et al., 2020; Schmidts et al., 2020)^1^ show that even emotional distraction is reduced when distractors are more frequent. These findings are commonly used to support the claim that the expectation of frequent distractors increases the benefits of proactive control, leading to improved performance.

But does it? A concern with distractor frequency manipulations is that increased distractor frequency is often confounded with greater experience. Selection history is thought to establish persistent biases in attentional deployment based on statistical regularities (Anderson et al., 2021; Awh et al., 2012; Geng et al., 2019). In support of this claim, distractors that appear in locations where distractors are more frequent are more easily ignored (Ferrante et al., 2018; Wang & Theeuwes, 2018a, 2018b). Similarly, the statistical regularity of distractor features has been shown to facilitate distractor avoidance (Kim et al., 2023; Stilwell et al., 2019; Vatterott & Vecera, 2012). A recent study by Kim and Anderson (2022) further suggests that selection history may prompt proactive distractor suppression. Learned contingencies of distractor presentation may therefore direct attentional selection instead of, or at least in addition to (Gao & Theeuwes, 2020), expectation of upcoming distraction.

One way to manipulate expectations without affecting distractor frequency is to cue upcoming distractors while holding their frequency constant. Contrary to an expectation-based account of proactive control, distraction is often unaffected (Augst et al., 2014; Schmidts et al., 2020) or even *worsened* (Kleinsorge, 2007, 2009) when negatively valenced distractors are explicitly cued. Some evidence further suggests that cues are similarly ineffective for the detection of threat related target stimuli (Aue et al., 2013, 2019).

While expectation of distractors should encourage more effective proactive cognitive control mechanisms (according to a cost/benefit analysis), there is little evidence that participants *can or do* make use of explicit expectation of emotional distractors. But explicit pre-trial cues may be poorly suited to eliciting proactive control. Cue-to-stimulus interval, motivation, and conflict frequency may alter the likelihood of engaging in effective cognitive control in response to conflict cues, regardless of expectation (Bugg & Smallwood, 2016; Chiew & Braver, 2016; Goldfarb & Henik, 2013; Marini et al., 2015). Cues themselves may also induce distraction, possibly drawing attention to distractor locations or priming distractor identity, impairing task performance (Moher & Egeth, 2012; see also Chelazzi et al., 2019). Thus, explicit cues may not be the best way to test whether expectations encourage the use of proactive control.

Here we use a different approach. The aim of the studies reported here is to investigate whether expectations of distraction, when divorced from experience, induces the use of proactive control. In three experiments, participants completed a simple letter identification task while ignoring task-irrelevant emotionally negative or neutral images. Instead of using explicit cues, we manipulated expectation implicitly. Distractors were presented either predictably (on every fourth trial) or randomly on 25% of trials, allowing us to manipulate expectation of upcoming distractors while holding the overall frequency of distractors constant.

## Experiment 1

The goal of Experiment 1 was to assess whether manipulations of expectation, while holding the frequency of distractors constant, can reduce distraction by task-irrelevant emotional images. Participants were asked to complete a simple letter identification task while emotional and neutral images from the International Affective Picture System (IAPS; Lang et al., 2008) were presented in the periphery on 25% of trials. To avoid the methodological concerns raised by explicit cues, expectation was manipulated by ordering of trials. Distractors occurred on every fourth trial (the predictable condition) or on a random 25% of trials (the unpredictable condition).

Replicating previous work, emotional images are expected to elicit greater distraction than neutral images. As distractors occur equally often (25% of trials) across the predictable and unpredictable conditions, differences in distraction between conditions should be owed to differences in the predictability of distractors. If expectation of upcoming distractors encourages the use of proactive control, then distraction by predictable distractors is expected to be reduced relative to that caused by unpredictable distractors. Because proactive control is also thought to reduce emotional distraction (i.e., the extent to which emotional distractors are more disruptive than neutral distractors), we further expect less emotional distraction in the predictable condition than in the unpredictable condition. Conversely, if experience drives the use of proactive control, no difference is expected between conditions.

### Method

#### Participants

Participants were 104 female,^2^ first-year undergraduate students (aged 18–32 years, *M* = 18.69, *SD* = 2.35). All participants had normal or corrected-to-normal vision and were not currently taking medication for depression or anxiety disorders. All participants provided written informed consent and received credits for a research participation component of their first-year studies. Data were collected in 2019. This study received approval from the Human Ethics Committee of Victoria University of Wellington, New Zealand.

#### Sample size

This experiment was powered to detect a main effect of predictability. G*power analysis^3^ indicated that to achieve a moderate effect (Cohen’s f = 0.25, ${\eta }_{p}^{2}$= 0.06), a minimum sample size of 98 participants would be needed to attain a power of 80%. We chose a moderate effect size as it enables us to detect an effect smaller than those found for other factors that improve distraction, including frequency (Grimshaw et al., 2018; Murphy et al., 2020) and reward (Walsh et al., 2018, 2019, 2021). For counterbalancing purposes, the total sample size was increased to 104 participants (52 in each predictability condition). Three participants who did not provide demographic information and a further two participants who were found to not meet eligibility criteria were excluded and replaced.

#### Stimuli

Twelve neutral and 12 negative images (occupying 12 × 12 degrees of visual angle) from the IAPS database (Lang et al., 2008) were used. Arousal and valence ratings of the images were obtained from female norms using the 9-point Self-Assessment Manikin rating scale reported in Lang et al. (2008). The neutral images were scenes of people performing everyday activities, rated moderate in valence and low in arousal. Negative images were scenes of gore and mutilation, rated as lower (more negative) in valence and higher in arousal. Six additional images of each valence were used for practice trials. All images were presented in colour on a black background and were matched for luminance and contrast using the MATLAB Shine toolbox (Willenbockel et al., 2010). IAPS image numbers and average valence and arousal ratings for image sets are presented in the Online Supplementary Material (OSM; Table S1).

**Fig. 1:**
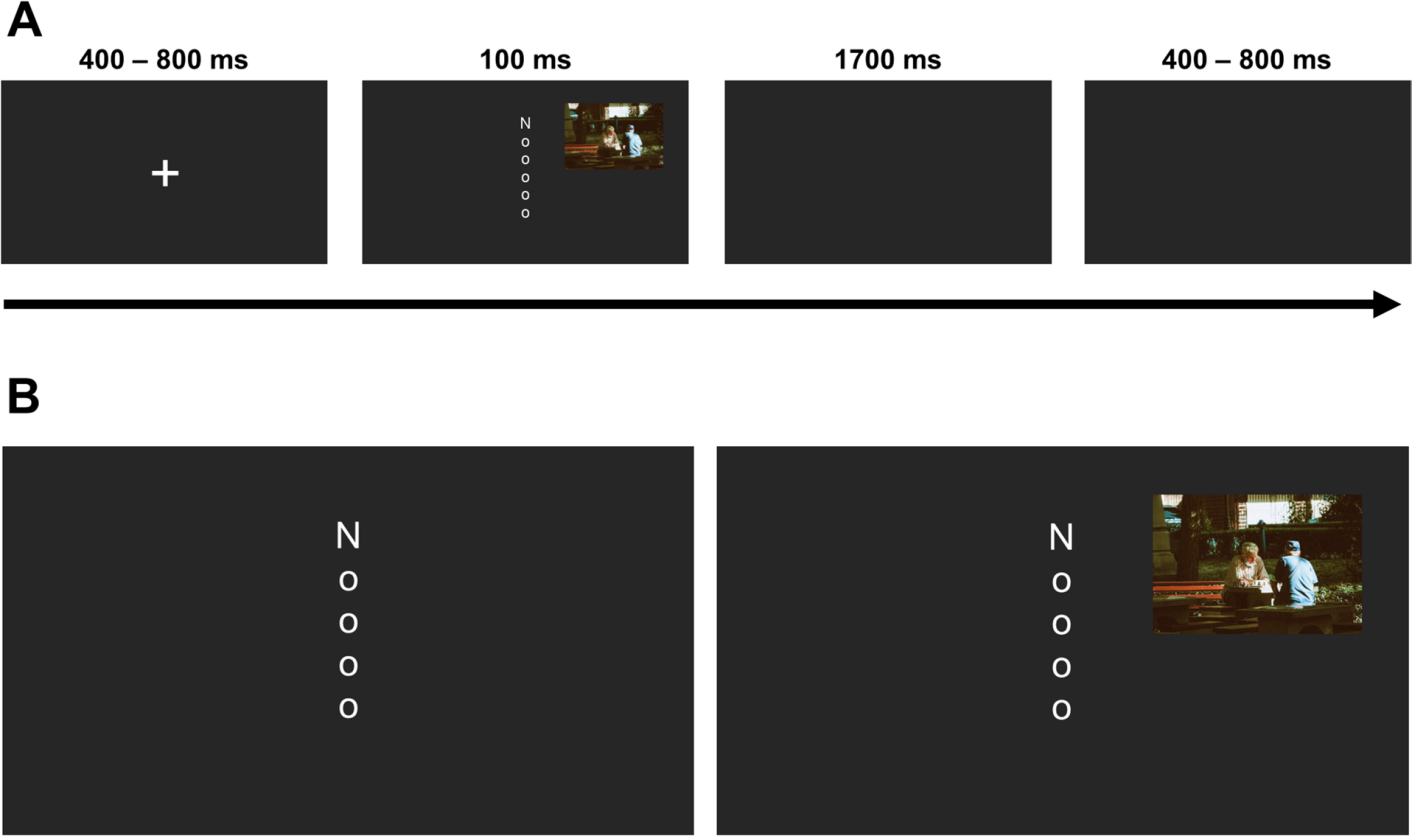
Trial structure of Experiment 1. (**A**) An example of a neutral distractor-present trial. (**B**) Examples of the target display for a distractor-absent trial (left) and a neutral distractor-present trial (right). Representative image sourced from https://www.pexels.com/@av-photography/ as IAPS images cannot be reproduced for this figure

The letter array consisted of five lower case o’s (occupying 0.22 by 0.22 degrees of visual angle each) and one uppercase N or K (occupying 0.69 by 0.69 degrees of visual angle), presented in white Arial font, arranged vertically along the midline of the screen. The letters were separated by 0.69 degrees of visual angle, with the top and bottom letters of the array positioned 1.75 degrees of visual angle from the center of the screen. On distractor-present trials, a single image (neutral or negative) was presented alongside the letter array in one of four quadrants, with the center of the image located 7.4 (horizontal) and 6.8 (vertical) degrees of visual angle from the center of the screen. Images appeared an equal number of times in each quadrant; an example of the task array including a distractor is shown in Fig. 1. These same stimulus displays have been used in our lab previously (Murphy et al., 2020), where they showed a typical distractor frequency effect; that is, they showed robust emotional distraction when images were presented on 25% of trials, and no distraction when presented on 75% of trials.

### Procedure

Participants were seated in a walled cubicle in a dimly lit room, 57 cm from a computer monitor (24-in., 1,920 × 1,080 pixels, AOC monitor with a refresh rate of 120 Hz), connected to a Dell T1700 computer. Viewing distance was maintained by a chinrest. Psychopy software (Peirce et al., 2019) was used for stimulus presentation and response recording. A trial began with the presentation of a central fixation cross (occupying 0.84 × 0.84 degrees of visual angle) which varied randomly in duration from 400 to 800 ms. Participants were then shown the letter array for 100 ms and were asked to indicate whether the letter K or N was present (using the index and middle fingers of their dominant hand on the 1 and 2 keys of the number-pad). Participants could respond within a 1,700-ms window from array onset. On participant response, the trial proceeded to a variable intertrial interval (ITI) equal to the duration of the fixation cross. On an incorrect or missing response participants received auditory feedback (a 100-ms tone). The button-response mapping was counterbalanced across participants. Within a block of trials, each target letter (K or N) occurred on a random half of trials and appeared equally often in each of the six array locations.

Participants were assigned to either the predictable condition, wherein distractors occurred on every fourth trial of the experiment (25% of trials), or to the unpredictable condition, wherein distractors occurred on a random 25% of trials. The experiment consisted of four blocks of 48 trials. Distractor valence was blocked so that all the distractors in a block were of the same valence, meaning that participants were able to expect the type of distractors that would appear. Distractors appeared once per block. Half the participants in each condition completed the blocks in a Neutral, Negative, Negative, Neutral order, and half did so in the inverse order. Blocks were separated by a self-paced break (30 s minimum). Participants also completed two 24-trial practice blocks. The first presented only neutral distractors while the second presented only negative distractors. Distractors in these practice blocks were either predictable or unpredictable as per the participant condition. Pre-experiment instructions stated that an image would be present ‘on every fourth trial’ (the predictable condition) or ‘on some trials’ (the unpredictable condition). This information was presented through on-screen instructions and was also repeated verbally by the experimenter.

#### Data processing

Data were analyzed using R version 4.2.2. (R Core Team, 2022). All trials without a response or with a response time (RT) of less than 200 ms were excluded from analysis (0.28% of trials). Mean RTs for correct trials were calculated in each condition, and a distraction index was calculated by subtracting the RT for distractor-absent trials from the RT of distractor-present trials in the same block. Accuracy was calculated as a percentage of correct trials. Accuracy difference scores were similarly calculated by subtracting the percentage of correct distractor-absent trials from the percentage of distractor-present trials in the same block. Two exclusion criteria were set prior to analysis, excluding participants with lower than 70% accuracy on blocks of either valence, or accuracy lower than 75% overall. No participants met these criteria. Effect sizes are reported as Cohen’s d_z_ for within-subject comparison and d_s_ for between subject comparisons, or as ${\eta }_{p}^{2}$.

### Results and discussion

#### Response times (RTs)

**Fig. 2:**
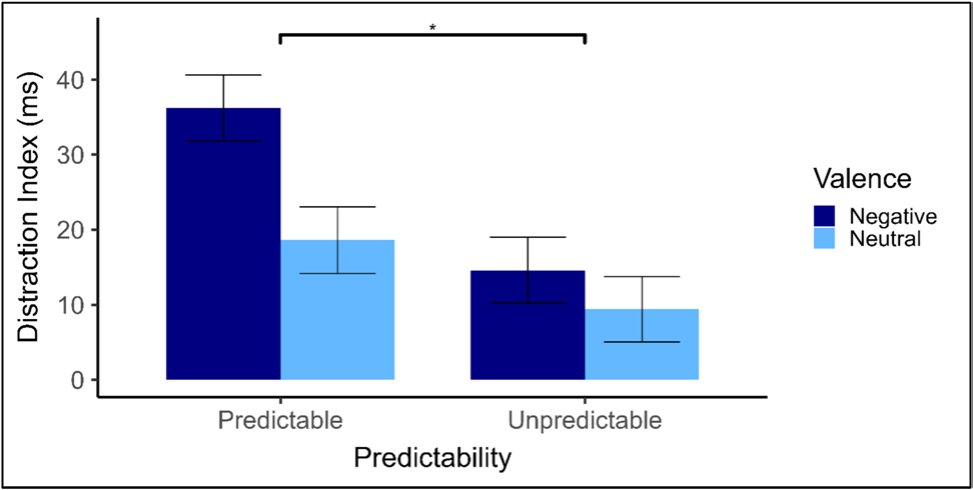
Experiment 1 mean distraction indices. Error bars indicate the standard error of the mean corrected for within-subjects comparisons (Morey, 2008). * p < .05. **p < .01. ***p < *.001*

Table I presents the mean RTs and standard deviations by Predictability, Valence and Distractor presence conditions, along with distraction indices. Paired-samples t-tests revealed that distraction is present for each valence of distractor within each condition (all *p* < 0.05), indicating that distractor presence is detrimental to task performance regardless of valence. To assess whether expectation of the upcoming distractor reduced distraction, distraction indices were entered into a 2 (valence: neutral, negative) × 2 (predictability: predictable, unpredictable) mixed ANOVA. A main effect of valence was observed, *F*(1, 102) = 12.28, *p* < 0.001, ${\eta }_{p}^{2}$ = 0.11, in which negative distractors (*M* = 27 ms, *SD* = 43) elicited greater distraction than neutral distractors (*M* = 12 ms, *SD* = 29). Together, these findings confirm that the modified task-irrelevant emotional distractor paradigm was able to elicit emotional distraction (as illustrated in Fig. 2).

**Table 1:** Mean (SD) response time (RT), Distraction indices in milliseconds and paired-samples t-test results, including confidence intervals (CIs) and effect sizes (Cohen’s d_z_), by Valence, Predictability and Distractor presence in Experiment 1

	Present	Absent	Distraction index	95% CI		t	d_z_
				LL	UL		
Predictable							
Negative	552 (77)	516 (55)	36 (48)	22.78	49.67	5.41***	0.75
Neutral	532 (67)	517 (59)	15 (27)	7.14	22.09	3.92***	0.54
Unpredictable							
Negative	532 (77)	513 (62)	19 (35)	8.97	28.24	3.88***	0.54
Neutral	524 (69)	514 (66)	9 (31)	0.75	18.13	2.18*	0.30

Distraction index = RT (distractor-present) – RT (distractor-absent).

The t-values and effects sizes present in this table are derived from paired-samples t-tests, comparing distractor-absent to distractor-present trials.

* p < 0.05. **p < 0.01. ***p < *0.001*

A main effect of predictability was also observed, *F*(1, 102) = 4.19, *p* = 0.043, ${\eta }_{p}^{2}$ = 0.04, in which distraction indices were lower in the unpredictable *(M* = 14 ms, *SD* = 33) than in the predictable condition (*M* = 25 ms, *SD* = 40). Thus, distraction was paradoxically *greater* when participants could predict distractor occurrence. Finally, the Predictability × Valence interaction was non-significant, *F*(1,102) = 2.01, *p* = 0.159, ${\eta }_{p}^{2}$ = 0.02, indicating that the paradoxical effect of expectation did not depend on distractor valence.

### Accuracy

Participants’ mean accuracy scores by Predictability, Valence, and Distractor presence are presented in Table I, along with accuracy distraction indices. Accuracy was high overall, with participants responding incorrectly to the letter task on only 5.6% of trials on average. As shown in Table I, none of the conditions produced significant differences in accuracy between distractor present and distractor absent trials. To assess whether participants strategically slowed responding on distractor present trials to increase the accuracy of their responses, accuracy difference scores were entered into a 2 (valence: neutral, negative) × 2 (predictability: predictable, unpredictable) mixed ANOVA which revealed no significant effects. There is therefore no evidence that participants in either condition selectively slowed responding in favor of accuracy.

**Table 2:** Mean accuracy (SD) in % correct trials, accuracy difference, and paired-samples t-test results, including confidence intervals (CIs) and effect sizes (Cohen’s d_z_), by Valence, Predictability and Distractor presence in Experiment 1

	Present	Absent	Difference	95% CI		*t*	*d*_*z*_
				LL	UL		
Predictable							
Negative	95.8 (5.3)	94.6 (4.6)	1.2 (5.9)	−0.48	2.80	1.42	0.20
Neutral	96.1 (4.8)	95.0 (4.2)	1.1 (4.6)	−0.15	2.42	1.78	0.25
Unpredictable							
Negative	93.0 (7.9)	93.9 (5.7)	−1.0 (7.6)	−3.06	1.16	0.90	0.13
Neutral	94.2 (5.3)	93.5 (5.1)	0.6 (4.7)	−0.68	1.94	0.96	0.13

The t-values and effects sizes present in this table are derived from paired-samples t-tests, comparing accuracy on distractor-absent to distractor-present trials.

* p < 0.05. **p < 0.01. ***p < *0.001*

#### Summary

The current findings provide no evidence that expectation of upcoming distractors reduces distraction, consistent with some previous cue-based studies (Kleinsorge, 2009; Schmidts et al., 2020). Indeed, expectation actually *increased* distraction, though we did not find this paradoxical effect of expectation to be unique to negative stimuli. In sum, our findings therefore suggest that expectation alone does not induce proactive control of attention.

## Experiment 2

The goal of Experiment 2 was to replicate and extend the findings of Experiment 1 while making two important methodological changes. The first was the addition of an unpredictable high-frequency condition, in which distractors occurred on a random 75% of trials. This condition allows us to confirm that distraction is in fact attenuated in this paradigm when distractors appear frequently (i.e., using a frequency manipulation similar to that in Grimshaw et al., 2018; Murphy et al., 2020). The unpredictable low-frequency condition could then be compared to the unpredictable high-frequency condition (which differs in frequency but not predictability) and to the predictable low-frequency condition (which differs in predictability but not frequency).

The second change was that distractors were presented at fixation, flanked by the target letters. Distractors in Experiment 1 were presented in predictable order but could still appear in any of four locations. Hence, distractor *location* was still unpredictable. It is possible that expectation of a specific upcoming distractor location is necessary to pre-emptively suppress attention to a distractor. We note also that central distractors primarily index the ability to disengage attention, while peripheral distractors both attract and hold attention. These factors should make it easier for participants to use proactive control to reduce distraction. We also presented a pixel-scrambled image on distractor absent trials to equate visual stimulation across all conditions. Iterations of the emotional task-irrelevant distractor paradigm using centrally presented distractors have previously been shown to induce robust emotional distraction (Padmala et al., 2017; Schmidts et al., 2020; Walsh et al., 2018, 2019).

If expectation encourages the use of proactive control mechanisms, we expect that there should be less distraction in the predictable low-frequency and unpredictable high-frequency condition than in the unpredictable low-frequency condition. However, if proactive control is instead encouraged by experience (i.e., frequent exposure to distractors), we expect less distraction in the unpredictable high-frequency condition than in either the predictable or unpredictable low-frequency conditions. Lastly, we again expect these effects to interact with valence.

### Method

#### Participants

Participants were 96 female, first-year undergraduate students (aged 18–30 years, *M* = 19.24, *SD* = 2.24). All participants had normal or corrected-to-normal vision and were not currently receiving treatment for depression or anxiety disorders. All participants provided written informed consent and received credits toward their first-year studies. One participant withdrew from the study due to discomfort and was replaced. Data were collected in 2020. This study received approval from the Human Ethics Committee, Victoria University of Wellington (New Zealand).

#### Sample size

We powered Experiment 2 to be able to detect a Frequency × Valence interaction, that is, a reduction in emotional distraction when distractors appeared frequently. We reasoned that if expectations cause similar effects to frequency, we should see a similar effect size. Based on an unpublished study using central distractors with a frequency manipulation (Grimshaw et al., 2020), we estimated an effect size of Cohen’s f = 0.33 (η_p_^2^ = 0.10). G*power analysis indicated that a sample size of 93 participants provides power of 80%.^4^ For counterbalancing purposes a sample of 96 participants (32 per condition) was recruited.

#### Stimuli

The same IAPS images from Experiment 1 were used. Distractors were presented at fixation, flanked above and below by the letter array. For each IAPS image, three pixel-scrambles were made. The pixel-scrambled images were of the same size and resolution as the original intact image and were presented on distractor-absent trials (now called scrambled-distractor trials, in contrast to intact-distractor trials). Each of the 12 intact IAPS images were shown once per block of 48 trials in the unpredictable low-frequency and predictable low-frequency conditions. In the unpredictable high-frequency condition, each intact image was shown three times per block, with the constraint that the same images were not presented consecutively. On scrambled trials a single scramble of each of the 12 intact images was used in the high-frequency condition while three scrambles of each intact image were used in the low-frequency conditions. Scrambled images were only ever presented in the same block as their intact counterparts. All images were presented in colour, occupying 11 × 8.25 degrees of visual angle, on a black background and were matched for luminance using the MATLAB Shine toolbox. The letter array consisted of six letters arranged in two rows of three, positioned 4.87º above and below the midline of the screen and horizontally separated by 5.07º. The letters of the letter array were all presented in white Arial front occupying 0.86º × 0.92 º of visual angle. Five of the letters in the array were capital O’s while a random sixth letter was either a capital K or N.

#### Procedure

**Fig. 3:**
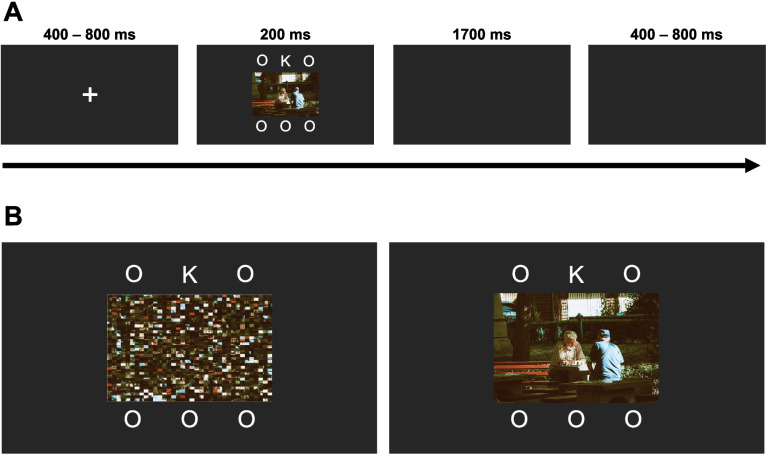
Trial structure of Experiment 2. (**A**) An example of a neutral distractor-present trial. (**B**) Examples of the task array for a scrambled-distractor trial (left) and a neutral intact-distractor trial (right). Representative image sourced from https://www.pexels.com/@av-photography/as IAPS images cannot be reproduced for this figure

Experiment 2 made use of the same testing environment and equipment as Experiment 1. Participants were randomly assigned to one of three conditions. In the unpredictable high-frequency condition, intact distractors were present on a random 75% of trials. In the unpredictable low-frequency condition intact distractors were present on a random 25% of trials. In the predictable low-frequency condition intact distractors were presented on every fourth trial of a block (25% of trials overall). On scrambled-distractor trials a pixel-scrambled image was instead presented in the same central location (see Fig. 3).

The experimental task was identical to Experiment 1 except that the task display was now presented for 200 ms. Participants were informed that all trials would contain an image, that the image was either intact or scrambled, and the percentage of trials that would contain an intact distractor. They were also informed that “an intact image would occur on every fourth trial” (in the predictable low-frequency condition) or that the order of presentation of scrambled and intact images would be random (in the unpredictable high-frequency and unpredictable low-frequency conditions). Participants again completed two blocks of practice trials (24 trials each). Distractor frequency in these practice blocks matched that of the participants’ assigned condition in the main body of the experiment.

Following the experiment, participants were given a questionnaire assessing their awareness of the frequency and predictability manipulation. This questionnaire asked: (i) the frequency of intact images as a percentage), (ii) whether it felt as if the images were presented randomly or in order, (iii) whether they were able to predict upcoming intact distractors, and (iv) whether they used some strategy to prepare for intact distractor images. If they answered yes to the fourth question, they were also asked (v) to detail what that strategy was. The questionnaire was accompanied by verbal instructions and the chance to ask for clarification.

#### Data processing

Data were analyzed using R version 4.2.2. (R Core Team, 2022). Tukey’s HSD tests were conducted in Jamovi version 2.3.26.0 (The Jamovi Project, 2024). All trials without a response or with an RT of less than 200 ms were excluded from all analyses (0.36% of trials). RTs, distraction indices, accuracies and accuracy differences were calculated as in Experiment 1. Two preregistered exclusion criteria were established, excluding participants with lower than 70% accuracy on blocks of either valence, or accuracy lower than 75% overall. Two participants (one from the unpredictable high-frequency condition and one from the predictable low-frequency condition) obtained an overall accuracy of less than 75% and were removed from the analysis. Effect sizes are reported as Cohen’s d_z_ for within-subject comparison and d_s_ for between-subject comparisons, or as η_p_^2^.

### Results and discussion

#### RTs

**Fig. 4:**
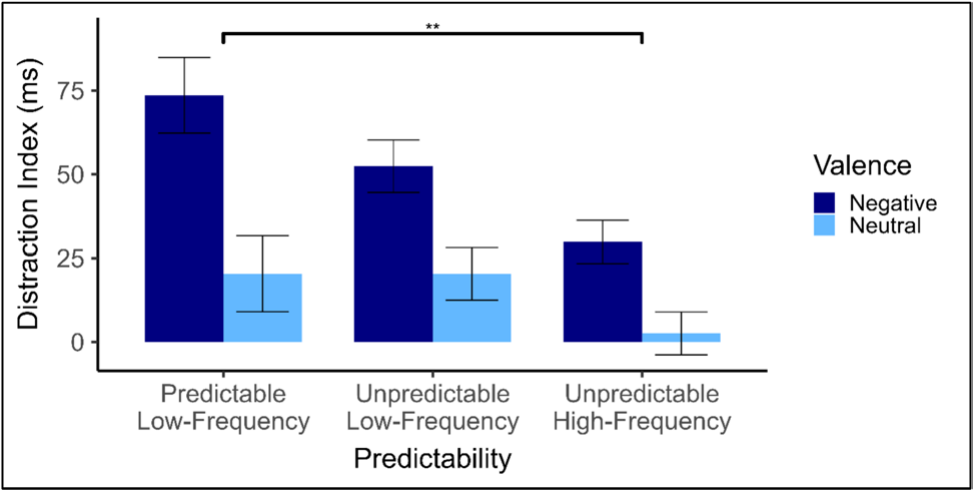
Experiment 2 mean distraction indices*.* Error bars indicate the standard error corrected for within-subjects comparisons (Morey, 2008). * p < .05. **p < .01. ***p < *.001*

Mean RTs by Predictability, Valence, and Distractor type, along with distraction indices are shown in Table I. Paired-samples t-tests indicated that RTs were significantly slower on trials containing an intact (vs. scrambled) distractor in all conditions except for neutral distractors in the unpredictable high-frequency condition. Thus, the paradigm reliably produced distraction by intact images. Distraction indices were entered into a 2 (valence: neutral, negative) × 3 (predictability condition: predictable low-frequency, unpredictable low-frequency, unpredictable high-frequency) mixed ANOVA. Analysis revealed a main effect of distractor valence, *F*(1,91) = 27.27, *p* < 0.001, *η*_*p*_^*2*^ = 0.23. Distraction was greater in blocks containing negative distractors (*M* = 52 ms*, SD* = 69) than in blocks containing neutral distractors (*M* = 14 ms*, SD* = 35). Analysis also revealed a main effect of predictability, *F*(2,91) = 4.71, *p* = 0.011, *η*_*p*_^*2*^ = 0.09. A Tukey’s HSD test found that there was more distraction in the predictable low-frequency condition than unpredictable high-frequency condition, *p* = 0.009. Distraction in the unpredictable low-frequency condition fell between these two conditions but did not differ significantly from either the unpredictable high-frequency condition, *p* = 0.127, or the predictable low-frequency condition, *p* = 0.551 (see Fig. 4). There were no significant interactions involving valence.

**Table 3:** Mean (SD) response times (RTs), Distraction indices in milliseconds, and paired-samples t-test results, including confidence intervals (CIs) and effect sizes (Cohen’s d_z_), by Valence, Predictability and Distractor type in Experiment 2

	Intact	Scrambled	Distraction index	95% CI		*t*	*d*_*z*_
				LL	UL		
Predictable low-frequency							
Negative	689 (128)	615 (60)	74 (93)	40.18	107.03	4.49***	0.79
Neutral	631 (71)	611 (59)	20 (35)	7.71	32.87	3.29**	0.58
Unpredictable low-frequency							
Negative	681 (101)	629 (82)	52 (60)	30.40	74.35	4.87***	0.87
Neutral	633 (79)	613 (84)	20 (33)	8.12	32.46	3.41**	0.61
Unpredictable high-frequency							
Negative	679 (113)	649 (100)	30 (34)	17.51	42.14	4.95***	0.89
Neutral	648 (94)	646 (97)	3 (36)	−10.50	15.67	0.40	0.07

Distraction index = RT (distractor-present) – RT (distractor-absent).

The t-values and effects sizes present in this table are derived from paired-samples t-tests, comparing RT on intact-distractor trials to scrambled-distractor trials.

* p < 0.05. **p < 0.01. ***p < *0.001*

Overall, we find no evidence of improved performance when low-frequency distractors are predictable. The findings of Experiment 2 differed from that of Experiment 1 in that we did not statistically replicate the paradoxical increase in distraction for predictable over unpredictable distractors, although the effect was in the same direction and of a similar magnitude.

#### Accuracy

Overall accuracy scores are shown in Table I. Participants responded incorrectly to the letter task on 7.5% of trials on average. Accuracy difference scores were entered into a 2 (valence: neutral, negative) × 3 (predictability: predictable low-frequency, unpredictable low-frequency, unpredictable high-frequency) mixed ANOVA. This analysis found only a significant effect of predictability, *F*(2,91) = 3.22, *p* = 0.044, *η*_*p*_^*2*^ = 0.07. Reflecting the pattern seen in the RT distraction indices, a subsequent Tukey’s HSD test indicated that intact distractors produced greater impairment in the predictable low-frequency condition (Difference = −3.54%, *SD* = 8.70) than in the unpredictable high-frequency condition (Difference = −0.33%, *SD* = 6.36), *p* = 0.038. Scores in the unpredictable low-frequency condition did not differ significantly from either condition. This similar pattern of results indicates that participants were not sacrificing speed for accuracy. As in Experiment 1, we find no difference between the predictable low-frequency and unpredictable low-frequency conditions, indicating that expectation did not facilitate (or statistically impair) performance on the letter task.

**Table 4:** Mean (SD) accuracy scores in % correct trials, and paired-samples t-test results, including confidence intervals and effect sizes (Cohen’s dz), by Valence, Predictability Condition, and Distractor type in Experiment 2

	Intact	Scrambled	Difference	95% CI		*t*	*d*_*z*_
				LL	UL		
Predictable low-frequency							
Negative	89.5 (11.4)	94.2 (5.0)	−4.7 (10.5)	−8.48	−0.91	2.53*	0.45
Neutral	91.4 (7.2)	93.8 (4.8)	−2.4 (6.4)	−4.69	−0.09	2.12*	0.37
Unpredictable low-frequency							
Negative	90.8 (7.6)	93.2 (5.1)	−2.4 (7.2)	−5.03	0.22	1.87	0.34
Neutral	92.6 (5.7)	93.0 (5.1)	−0.4 (5.8)	−2.57	1.69	0.42	0.08
Unpredictable high-frequency							
Negative	90.9 (6.0)	90.9 (7.8)	0.02 (6.3)	−2.29	2.33	0.02	0.003
Neutral	92.0 (5.1)	92.7 (6.9)	−0.7 (6.5)	−3.07	1.70	0.59	0.11

The t-values and effects sizes present in this table are derived from paired-samples t-tests, comparing accuracy on intact-distractor trials to scrambled-distractor trials

* p < 0.05. **p < 0.01. ***p < *0.001*

#### Post-experiment questionnaire

To determine whether participants were aware of the frequency and predictability with which distractors appeared, we examined their responses on the post-experiment questionnaire. Across conditions, most participants (82%) responded correctly when asked what percentage of trials contained an intact distractor image. Importantly, participants in the predictable low-frequency condition responded correctly most often (91%), although only 72% indicated that the distractors appeared in a predictable order, even though this fact was stated explicitly during task instructions. Fewer participants correctly reported the proportion of distractors in the unpredictable conditions (88% in the low-frequency condition; 69% in the high-frequency condition). A substantial portion of participants also indicated that distractors were presented in a fixed order in the unpredictable high-frequency condition (31%), with fewer (6%) in the unpredictable low-frequency condition. Taken together these findings suggest that participants in the predictable low-frequency condition had the clearest explicit awareness of the contingency.

As for participant intuition about upcoming distractors, few stated that they could predict when an upcoming distractor would appear in the unpredictable conditions (high-frequency = 9%, low-frequency = 6%). Substantially more participants reported that they were able to predict distractors in the predictable low-frequency condition (69%). Participants in the predictable low-frequency condition also indicated most often (28%) that they used some sort of strategy to avoid distraction on upcoming trials (unpredictable high-frequency = 9%, unpredictable low-frequency = 22%). Of the 18^5^ participants who reported that they employed some sort of strategy, most (13 responses) described a strategy detailing avoidance of the distractors. Notably, only three participants (all from the predictable low-frequency condition), alluded to proactive preparation for upcoming intact-distractor trials. Only two participants indicated that they attempted to prioritize task relevant stimuli. Thus, while some participants were aware of the contingencies, they appeared unable (or unwilling) to use this knowledge to improve performance, and very few reported using specific strategies intentionally. A table detailing the number of participants who indicated each type of strategy is presented in OSM Table S2.

#### Exploratory analyses

As the above analyses suggest no benefit for predictability, an alternative experience-based modification of control warrants some investigation. If experience facilitates control, we posit that some experience is first needed for control to be modified. Hence, there should be some indication of improvement over trials in the unpredictable high-frequency condition which should be missing from the low-frequency conditions. To assess whether cognitive control improved as experience was acquired, the distraction indices for the first block of each distractor valence were compared to the second (as in Experiment 1, two blocks of trials are presented for each valence). An exploratory 2 (valence: neutral, negative) × 2 (block: first, second) × 3 (predictability: predictable low-frequency, unpredictable low-frequency, unpredictable high-frequency) mixed ANOVA was conducted. Analyses indicated no effect of block nor any interactions, counter to what would be expected if participants improved over time. Exploratory investigation into the effect of experience provided no support for reduction of distraction over time, although the experiment was not optimally designed to track trial-by-trial changes in distraction. It is possible that experience-based modifications in task performance were very quickly implemented, possibly over the training trials, which were unfortunately not recorded. A future study, designed to assess the change in distraction over time, is required to probe this learning process further.

To determine whether we replicated the previously reported effect of distractor frequency (Grimshaw et al., 2018; Schmidts et al., 2020), we compared distraction indices between only the unpredictable low-frequency and high-frequency conditions. A 2 (valence: neutral, negative) × 2 (distractor frequency: low, high) mixed ANOVA indicated main effects of both frequency,* F*(1,60) = 6.38, *p* = 0.014,* η*_*p*_^*2*^ = 0.10, and valence,* F*(1,60) = 17.29, *p* < 0.001, *η*_*p*_^*2*^ = 0.22, reflecting greater distraction when distractor frequency was low (*M* = 36 ms, *SD* = 51) relative to high (*M* = 16 ms, *SD* = 37) and when distractors were negative (*M* = 41 ms, *SD* = 49) as opposed to neutral (*M* = 11 ms, *SD* = 35). However, no Frequency × Valence interaction was found, *F*(1,60) = 0.12, *p* = 0.735, *η*_*p*_^*2*^ = 0.002. Notably, proactive control appears to be induced in the high-frequency condition. Our findings are therefore in line with previous studies, although the effect of frequency was not specific to emotional distractors.

It is possible that reduced distraction in the high-frequency condition is owed not to improved control but instead to the repetition of distractor images in the high-frequency condition. Distractors were repeated three times per block (as opposed to being presented only a single time in the low-frequency condition), possibly leading to a habituation in the response to these stimuli. Event-related potentials which are associated with motivated attentional engagement (the N200 and LPP) have, however, been shown to be robust to stimulus repetition, and particularly so when the stimulus repetitions are spaced, suggesting that emotional stimuli likely remain distracting even after many repetitions (Ferrari et al., 2017). Although we cannot rule out an effect of specific stimulus repetition, we suggest that the difference in task performance between conditions derives from the response to the distracting emotional content of the stimuli. Notably, improved control as a result of stimulus repetition still points to an important role for experience, and not expectation, in encouraging the use of proactive control.

#### Summary

In Experiment 2 we used central distractors to ensure that distractor location was 100% predictable. Central distractors also tap largely into the ability to disengage attention, as opposed to peripheral distractors which both attract and hold attention. Both factors should have made it easier for participants to implement proactive control. And yet, findings in Experiment 2 replicated those of Experiment 1 in that expectation still did not decrease distraction. Indeed, the presence of predictable distractors impaired performance numerically, in both RT and accuracy, although not significantly. At the same time, we confirmed that increased distractor frequency *does* reduce distraction using the central distractor paradigm. Following from this, the present findings suggest that participants in the predictable low-frequency condition are not implementing control in a way that takes the predictable presentation of distractors into account. Again, these findings lend support to the conclusion that trial-by-trial expectation of distractors alone does not elicit proactive control.

The post-experiment questionnaire responses suggest that participants did not perfectly retain the statistical regularities of the task (distractor frequency and order of presentation). Critically though, awareness of the task contingencies was best in the predictable low-frequency condition (where distraction was greatest) while awareness was worst in the unpredictable high-frequency condition (where distraction was lowest). This pattern of findings further suggests that expectation does not prevent distraction.

## Experiment 3

An alternative explanation for the findings of Experiments 1 and 2 is that the cost of implementing more effortful proactive control strategies, especially on a trial-by-trial basis, discourages their use. When distractors are infrequent there is little incentive to switch to proactive control strategies since the benefit for these strategies is not often realized, regardless of how predictable distractors may be. Conversely when distractors are frequent the benefit of proactive control mechanisms is more obvious, as distraction is more often avoided. The findings of Experiments 1 and 2 may therefore reflect low motivation to implement cognitively demanding cognitive control strategies in the predictable condition. The goal of Experiment 3 was to assess whether expectation can be used to avoid distraction when people are provided with additional motivation to perform well on the letter task.

Previous task-irrelevant distractor studies indicate that distraction is reduced when participants are provided with either monetary (Padmala et al., 2017; Walsh et al., 2018, 2019) or even purely symbolic rewards (Walsh et al., 2021) for good performance. Participants were therefore randomly assigned to one of four conditions: seeing predictable or unpredictable distractors either with or without rewards for good task performance. If expectation *can* be used proactively, but participants are not usually motivated to do so, then we expect that participants in the predictable condition will engage in more effective proactive control when rewarded for good performance on the letter task. That is, they will use their expectations to shift to proactive control when the benefits are increased relative to the costs.

### Method

#### Participants

Participants were 144 first-year undergraduate students (79 female, 65 male) (aged 18–25 years, *M* = 19, *SD* = 1.3). Both men and women were recruited in this study to increase the generalizability of our findings. All participants had normal or corrected-to-normal vision and were not currently taking medication for depression or anxiety disorders. All participants provided written informed consent and received credits for a research participation component of their first-year studies. Data were collected in 2021. This study received approval from the Human Ethics Committee of the School of Psychology, Victoria University of Wellington, New Zealand.

#### Sample size

We aimed to recruit a sample large enough to detect an interaction between expectation of distraction and distractor valence (similar to the distractor frequency × valence interaction in previous studies) in either the rewarded or unrewarded condition. G*Power analysis indicated a total sample size of 144 (36 per condition) would be sufficient to detect an effect size of ${\eta }_{p}^{2}$ = 0.10 (Cohen’s *f* = 0.34) with 80% power.

#### Stimuli

Three image sets were used in this experiment. The first was a set of 12 neutral images which were used as distractors in a baseline block of trials. The second was the same set of images used in previous experiments which were shown only to female participants. A third set was made for male participants. This set was constructed to have similar valence and arousal ratings to the images used in Experiment 1 and Experiment 2.^6^ Mean valence and arousal ratings for these sets are listed in OSM Table S1. Stimulus displays were the same as those presented in Experiment 2.

#### Procedure

**Fig. 5:**
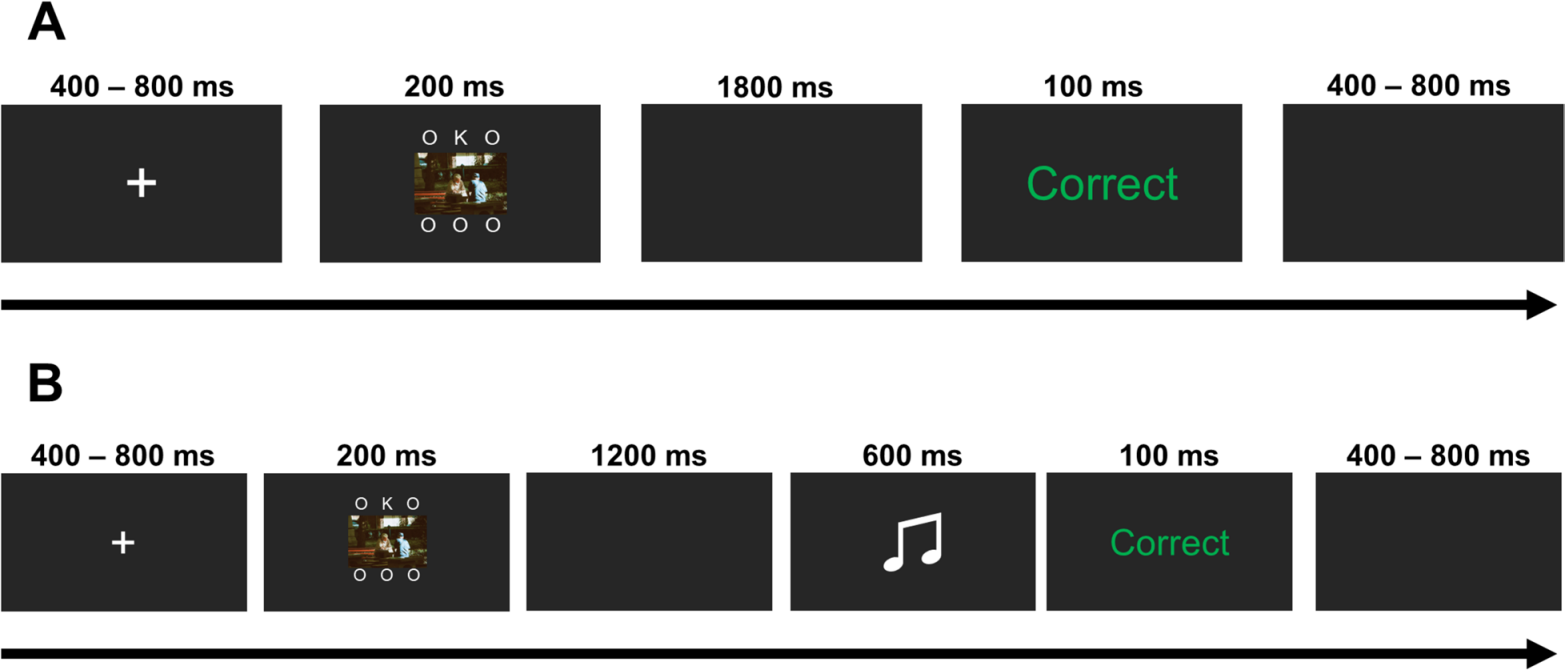
Trial structure of Experiment 3. (**A**) Progression of a trial in the baseline and unrewarded trials. (**B**) Progression of a rewarded trial. Representative image sourced from https://www.pexels.com/@av-photography/as IAPS images cannot be reproduced for this figure

Experiment 3 used the same apparatus as previous experiments and the central distractor task from Experiment 2 (see Fig. 5). Participants were randomly assigned to the predictable (low-frequency) or unpredictable (low-frequency) conditions which were identical to those in Experiment 2. In each predictability condition participants were then further assigned to be rewarded or not for their performance. Experiment 3 employed a 2(Predictability: Predictable, Unpredictable) × 2(Reward: Rewarded, Unrewarded) between-subjects design, with valence and distractor presence manipulated within-subjects.

All participants first completed 2 practice blocks (one containing only neutral distractors and one only negative) and a baseline block of trials containing only neutral images. Trials began with the presentation of a fixation cross for 400–800 ms, followed by the task display for 200 ms. Participants could then respond to the task display within an 1,800-ms response window. Feedback for task performance (in text) was then presented for 100 ms, informing participants that they were ‘Correct,’ ‘Incorrect’, or that they did not respond within the response window (“Please respond faster”). This was followed by an inter-trial interval of equal duration to the fixation cross. Importantly, in Experiment 3, the trial did not progress when participants responded. This was done to equate trial times in the rewarded and unrewarded conditions (see below). All participants completed one baseline block of 48 trials (with neutral distractors) and then progressed to four blocks of 48 experimental trials in which participants were either rewarded for their performance or not.

Participants in the unrewarded condition completed their experimental trials as in Experiment 2. Participants in the rewarded conditions were provided with symbolic ‘points’ and ‘levels’ for correctly responding to the letter task and doing so faster than the median RT of their baseline trials. Trials still began with a fixation cross and the task display but after participants responded they also received auditory feedback (a pleasant 600-ms jingle) when they were rewarded for that trial (1,200 ms after the task array onset), followed by the same feedback text as appeared in the baseline trials. For each rewarded trial a participant gained 10 points. At the end of each block of trials participants were shown which ‘level’ they had reached (depending on their point total), along with a pleasant animation of coins and a jingle. In the unrewarded conditions the task was identical to the baseline block trials. Between trial blocks, unrewarded participants were provided with on screen feedback for their performance (accuracy and average RT). All participants were also given feedback during an inter-block break as to their accuracy on the task. This feedback was included to further motivate good task performance.

#### Data processing

Data were analyzed using R version 4.2.2. (R Core Team, 2022). All trials without a response or with an RT of less than 200 ms were excluded from analysis (0.28% of trials). Mean RT, distraction indices, accuracies, and accuracy differences were calculated as in Experiment 1 and Experiment 2. Six participants met the exclusion criteria of lower than 75% accuracy on a block of trials. Three of these participants were assigned to the unrewarded unpredictable condition, two were assigned to the rewarded predictable condition and one had been assigned to unrewarded predictable condition. Final analyses are therefore based on 138 participants.

### Results and discussion

#### RTs

**Fig. 6:**
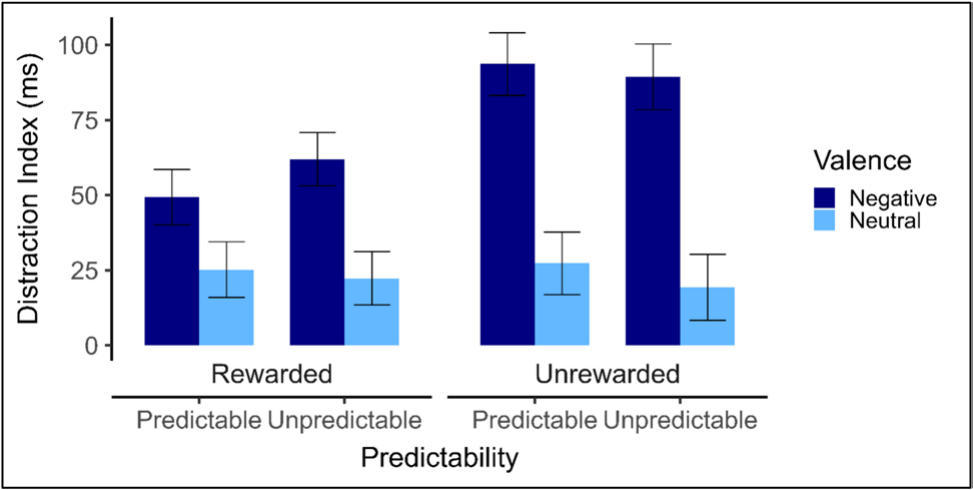
Experiment 3 mean distraction indices*.* Error bars indicate the standard error corrected for within-subjects comparisons (Morey, 2008)

Mean RTs by reward condition, predictability condition, distractor type and distractor valence are presented in Table I. Paired-samples t-tests indicate that intact distractors elicited significantly greater distraction than scrambles in all conditions. Mean distraction indices (see Fig. 6) were entered into a 2 (predictability: unpredictable, predictable) × 2 (distractor valence: negative, neutral) × 2 (reward: rewarded, unrewarded) mixed ANOVA. The only significant main effect was that of distractor valence, *F*(1,134) = 51.31, *p* < 0.001, ${\eta }_{p}^{2}$ = 0.28, indicating that distraction by negative distractors (*M* = 73 ms, *SD* = 90) was greater than that by neutral distractors (*M* = 24 ms, *SD* = 35). There was also a significant Reward × Valence interaction, *F*(1,134) = 6.75, *p* = 0.010, ${\eta }_{p}^{2}$ = 0.05. Follow-up Welch’s t-tests revealed that reward reduced distraction by negative images, (rewarded: *M* = 56 ms, *SD* = 79, unrewarded: *M* = 92 ms, *SD* = 99), *t*(127.93) = 2.35, *p* = 0.020, *d*_*z*_ = 0.4, but not distraction by neutral images (rewarded: *M* = 24 ms, *SD* = 29, unrewarded: *M* = 23 ms, *SD* = 41), *t*(119.45) = 0.04, *p* = 0.965, *d*_*z*_ = 0.01. Critically, the predicted interaction between reward and predictability was not observed, *F*(1,134) = 0.35, *p* = 0.55; that is to say, there is no evidence that rewards encouraged the use of expectation to avoid distraction. Indeed, there were no significant effects of predictability in either rewarded or unrewarded conditions.

**Table 5:** Mean (SD) response time (RT), Distraction indices in milliseconds and paired-samples t-test results, including confidence intervals (CIs) and effect sizes (Cohen’s d_z_), by Valence, Predictability, Reward and Distractor type in Experiment 3

	Intact	Scrambled	Difference	95% CI		t	d_z_
				LL	UL		
Rewarded Predictable							
Negative	633 (120)	583 (63)	49 (81)	21.03	77.62	3.55**	0.61
Neutral	589 (77)	564 (56)	25 (34)	13.22	37.18	4.28***	0.73
Rewarded Unpredictable							
Negative	645 (100)	583 (59)	62 (77)	35.97	87.90	4.84***	0.81
Neutral	593 (54)	571 (48)	22 (23)	14.50	30.04	5.82***	0.97
Unrewarded Predictable							
Negative	760 (162)	666 (82)	94 (101)	59.05	128.30	5.50***	0.93
Neutral	677 (91)	650 (75)	27 (37)	14.68	39.99	4.39***	0.74
Unrewarded Unpredictable							
Negative	768 (157)	679 (97)	89 (98)	54.78	123.99	5.26***	0.92
Neutral	688 (100)	668 (92)	19 (46)	2.99	35.55	2.41*	0.42

Distraction index = RT (distractor-present) – RT (distractor-absent).

The t-values and effects sizes present in this table are derived from paired-samples t-tests, comparing RT on intact-distractor and scrambled-distractor trials.

* p < 0.05. **p < 0.01. ***p < *0.001*

#### Accuracy

Mean accuracy scores are reported in Table I. Participants responded incorrectly to the letter task on 6.5% of trials on average. Accuracy difference scores were entered into 2(predictability: unpredictable, predictable) × 2(distractor valence: negative, neutral) × 2(reward: rewarded, unrewarded) mixed ANOVA. The ANOVA results indicated no significant main effects or interactions.

**Table 6:** Mean (SD) accuracy, accuracy difference, and paired-samples t-test results, including confidence intervals (CIs) and effect sizes (Cohen’s dz), by Valence, Predictability, Reward and Distractor presence in Experiment 3

	Intact	Scrambled	Difference	95% CI		*t*	*d*_*z*_
				LL	UL		
Rewarded Predictable							
Negative	89.8 (7.6)	91.9 (4.9)	−2.1 (6.3)	−4.26	0.16	1.89	0.32
Neutral	89.9 (6.3)	92.3 (4.4)	−2.4 (5.0)	−4.14	−0.63	2.76**	0.47
Rewarded Unpredictable							
Negative	87.8 (9.2)	90.2 (5.9)	−2.4 (9.6)	−5.64	0.83	1.51	0.25
Neutral	90.7 (7.6)	92.3 (3.9)	−1.6 (7.6)	−4.21	0.96	1.27	0.21
Unrewarded Predictable							
Negative	93.7 (5.4)	96.0 (3.3)	−2.3 (6.0)	−4.34	−0.26	2.29*	0.39
Neutral	96.3 (4.6)	97.1 (2.9)	−0.8 (4.9)	−2.48	0.87	0.98	0.17
Unrewarded Unpredictable							
Negative	91.6 (6.0)	96.2 (4.3)	−4.6 (7.2)	−7.12	−2.03	3.66***	0.64
Neutral	95.5 (5.6)	96.7 (2.6)	−1.2 (5.2)	−2.99	0.67	1.29	0.23

The t-values and effects sizes present in this table are derived from paired-samples t-tests, comparing reaction time on intact-distractor and scrambled-distractor trials.

* p < 0.05. **p < 0.01. ***p < *0.001*

#### Summary

Experiment 3 assessed whether participants would use expectations to motivate the use of proactive control when they were rewarded for good performance. Although reward was symbolic (points), the motivation manipulation was still effective, with less distraction by negative images when performance was rewarded. However, even with this increased motivation, participants did not use expectation of predictable distractors to improve performance.

## General discussion

Across three experiments we show that expectation of upcoming distraction does not make it easier to ignore emotional or non-emotional irrelevant images. Our findings are broadly in line with studies that have used cueing paradigms to manipulate expectations and which report that cues also do not prevent distraction (Augst et al., 2014; Schmidts et al., 2020; Wang & Theeuwes, 2018a) and may even be detrimental (Kleinsorge, 2007, 2009; Moher & Egeth, 2012). When distractors were infrequent, they produced high levels of distraction whether they were expected or not, suggesting that participants engage predominantly in reactive rather than proactive control when distractors are infrequent, regardless of expectations. We *do,* however, replicate (in Experiment 2) the finding (e.g., Grimshaw et al., 2018) that frequent distractors reduce distraction. Given that expectations alone do not appear to prevent distraction, this frequency effect more likely arises through increased experience provided by frequent distractors.

Although the use of proactive control when distractors are frequent may reflect intentional strategy, frequent distractors may alternatively elicit proactive control through implicit learning of statistical regularities within an experimental task without awareness of these underlying contingencies or intentional use of top-down strategies. Selection history is suggested to direct attention in just this way, for instance by inhibiting attention to locations where distractors frequently appear while prioritizing frequent target locations (Gao & Theeuwes, 2020; Wang & Theeuwes, 2018b). In Experiment 2 we found that our participants had some (though not perfect) knowledge of the contingencies (in part because we explicitly informed them), and yet were not able to use this knowledge to improve performance. In future research, it would be useful to omit these explicit instructions so the role of implicit learning could be more directly assessed.

Moreover, we note that Experiment 2 did not include a predictable high-frequency condition, which would have completed the factorial design and allowed us to assess interactions between frequency and predictability. Our primary rationale for including the unpredictable high-frequency condition was to allow us to compare the effect of frequency (the difference between the unpredictable high- and low-frequency conditions) to that of our expectation manipulation (the difference between the predictable and unpredictable low-frequency conditions). Under high-frequency conditions, expectation and experience are both high; it is therefore possible that further predictability would not affect performance. However, a previous study (Abado et al., 2020), shows that target selection can improve for threatening stimuli when the relative frequencies of threatening and neutral targets are manipulated. If expectation effects are context dependent in this way, then a future replication could include this fourth condition to determine whether the effects of frequency and expectation interact.

In Experiment 3, we tried to encourage participants to use predictive information by rewarding them (symbolically) for good performance. As expected, rewards did reduce distraction by negative images overall. However, this additional motivation did not affect the use of expectations to improve performance. An assumption that underlies the logic of predictability manipulations like ours (and cueing manipulations used by others) is that control is implemented on a trial-by-trial basis when distractors can be expected (Augst et al., 2014). Such transient implementation may be difficult, however, with its cost not offset even when rewards are available. Therefore, participants may still default to reactive control mechanisms. Indeed, a recent EEG study suggests that high distractor frequency elicits proactive control in a sustained manner across blocks, and not on a trial-by-trial basis (Murphy et al., 2020).

The finding of increased distraction in the predictable low-frequency conditions (significantly in Experiment 1, and numerically in Experiment 2) indicates that being able to expect distractors sometimes impairs performance. A possible explanation for this paradoxical finding is the attentional white bear effect; the involuntary allocation of attention to an expected task-irrelevant stimulus (Huffman et al., 2019; Lahav et al., 2012; Makovski, 2019; Makovski & Chajut, 2021; Moher & Egeth, 2012; Tsal & Makovski, 2006). If expected distractors involuntarily capture attention in this way, reorienting to the task-relevant stimuli would incur a cost to RT. However, we note that this paradoxical effect was not present in Experiment 3, in either the rewarded or unrewarded conditions.

An alternative explanation for our paradoxical distraction may be that the expectation manipulation introduces different working memory loads between the predictable and unpredictable low-frequency conditions, while participants in the predictable condition try to keep count of trials. We believe this is unlikely, however, because responses to the post study questionnaire in Experiment 2 do not suggest that participants used this information strategically.

## Conclusion

We find that expectation clearly does not reduce distraction, and may indeed increase it, particularly when distractors are emotional. Contrary to predictions from the DMC framework, expectations alone are insufficient to elicit proactive control of distraction, even when rewards are available for doing so. Obligatory attention to rare emotional (or at least threatening) people, objects, or events may well be adaptive. However, when they appear repeatedly, they lose informational value, and so it is also adaptive that we can *learn* to ignore them through experience. This dynamic response to emotional distractors illustrates the contextual adjustment of attention to emotional stimuli in line with both current goals and survival needs.

## Supplementary Information

Below is the link to the electronic supplementary material.

Supplementary file1 (DOCX 15 KB)

## Data Availability

All participants provided consent for de-identified aggregate data being stored online on the Open Science Framework (https://osf.io/kjgr9/).

## Footnotes

1 Task-irrelevant distractor tasks require attention to be directed to the task relevant stimulus (e.g., a simple letter detection task) while distractors are presented alongside the task on some trials. The difference in response time (RT) to the task as a factor of distractor presence (or the qualities of the distractors) is indicative of the distraction by the task irrelevant stimuli.

2 Differences in valence and arousal assessments of the negative IAPS images have previously been found between men and women (Lang et al., 2008). Given this difference, the present experiment included only female participants to ensure a more homogeneous interpretation of the images.

3 G*power analyses were conducted for a priori power analysis using the F-tests test family. For Experiment 1 we used ANOVA repeated-measures, between-factors as the statistical test. For Experiments 2 and 3 we used ANOVA repeated-measures, within-between interaction.

4 The power analyses for Experiments 2 and 3 were incorrectly reported in the preregistration for both experiments. These values have been corrected in the present document.

5 One additional participant indicated that they tried to focus primarily on the fixation cross between trials. As this was not related to the upcoming distractor present trials this response was not further considered. This response could have indicated an attempt to prioritize the distractor location (which appeared on fixation) or may indicate that the participant did not fully understand the question.

6 A distinct male image set was created to account for the difference in normative ratings of IAPS images between men and women.

## Code availability

Aggregate data and code used to conduct data collection for all studies are accessible at https://osf.io/kjgr9/

## Declarations

Ethics approval

### Open practices statement

Aggregate data and materials for all experiments are available at https://osf.io/kjgr9/. The hypotheses and analyses of Experiment 2 were preregistered (https://osf.io/s8jtr) as were those of Experiment 3 (10.17605/OSF.IO/AJEMH).

## References

[CR1] Anderson, B. A., Kim, H., Kim, A. J., Liao, M.-R., Mrkonja, L., Clement, A., & Grégoire, L. (2021). The past, present, and future of selection history. *Neuroscience & Biobehavioral Reviews,**130*, 326–350. 10.1016/j.neubiorev.2021.09.004 34499927 10.1016/j.neubiorev.2021.09.004 PMC8511179

[CR2] Abado, E., Sagi, J., Silber, N., De Houwer, J., Aue, T., & Okon-Singer, H. (2020). Reducing attention bias in spider fear by manipulating expectancies. *Behaviour Research and Therapy,**135*, Article 103729. 10.1016/j.brat.2020.103729 32980587 10.1016/j.brat.2020.103729

[CR3] Aue, T., Guex, R., Chauvigné, L. A. S., & Okon-Singer, H. (2013). Varying expectancies and attention bias in phobic and non-phobic individuals. *Frontiers in Human Neuroscience*, *7*. 10.3389/fnhum.2013.00418 10.3389/fnhum.2013.00418 PMC3737492 23964219

[CR4] Aue, T., Guex, R., Chauvigné, L. A. S., Okon-Singer, H., & Vuilleumier, P. (2019). Expectancies influence attention to neutral but not necessarily to threatening stimuli: An fMRI study. *Emotion,**19*(7), 1244–1258. 10.1037/emo0000496 30475038 10.1037/emo0000496

[CR5] Augst, S., Kleinsorge, T., & Kunde, W. (2014). Can we shield ourselves from task disturbance by emotion-laden stimulation? *Cognitive, Affective, & Behavioral Neuroscience,**14*(3), 1009–1025. 10.3758/s13415-013-0243-x 10.3758/s13415-013-0243-x 24399683

[CR6] Awh, E., Belopolsky, A. V., & Theeuwes, J. (2012). Top-down versus bottom-up attentional control: A failed theoretical dichotomy. *Trends in Cognitive Sciences,**16*(8), 437–443. 10.1016/j.tics.2012.06.010 22795563 10.1016/j.tics.2012.06.010 PMC3426354

[CR7] Botvinick, M. M., Braver, T. S., Barch, D. M., Carter, C. S., & Cohen, J. D. (2001). Conflict monitoring and cognitive control. *Psychological Review,**108*(3), 624–652. 10.1037/0033-295X.108.3.624 11488380 10.1037/0033-295x.108.3.624

[CR8] Braver, T. S. (2012). The variable nature of cognitive control: A dual-mechanisms framework. *Trends in Cognitive Sciences,**16*(2), 106–113. 10.1016/j.tics.2011.12.010 22245618 10.1016/j.tics.2011.12.010 PMC3289517

[CR9] Braver, T. S., Paxton, J. L., Locke, H. S., & Barch, D. M. (2009). Flexible neural mechanisms of cognitive control within human prefrontal cortex. *Proceedings of the National Academy of Sciences,**106*(18), 7351–7356. 10.1073/pnas.0808187106 10.1073/pnas.0808187106 PMC2678630 19380750

[CR10] Bugg, J. M., & Smallwood, A. (2016). The next trial will be conflicting! Effects of explicit congruency pre-cues on cognitive control. *Psychological Research Psychologische Forschung,**80*(1), 16–33. 10.1007/s00426-014-0638-5 25522873 10.1007/s00426-014-0638-5

[CR11] Burgess, G. C., & Braver, T. S. (2010). Neural mechanisms of interference control in working memory: Effects of interference expectancy and fluid intelligence. *PLoS ONE,**5*(9), Article e12861. 10.1371/journal.pone.0012861 20877464 10.1371/journal.pone.0012861 PMC2942897

[CR12] Carretié, L. (2014). Exogenous (automatic) attention to emotional stimuli: A review. *Cognitive, Affective, & Behavioral Neuroscience,**14*(4), 1228–1258. 10.3758/s13415-014-0270-2 10.3758/s13415-014-0270-2 PMC4218981 24683062

[CR13] Chelazzi, L., Marini, F., Pascucci, D., & Turatto, M. (2019). Getting rid of visual distractors: The why, when, how, and where. *Current Opinion in Psychology,**29*, 135–147. 10.1016/j.copsyc.2019.02.004 30856512 10.1016/j.copsyc.2019.02.004

[CR14] Chiew, K. S., & Braver, T. S. (2016). Reward favours the prepared: Incentive and task-informative cues interact to enhance attentional control. *Journal of Experimental Psychology. Human Perception and Performance*, *42*(1), 52–66. 10.1037/xhp0000129 10.1037/xhp0000129 PMC4688088 26322689

[CR15] Di Bello, F., Ben Hadj Hassen, S., Astrand, E., & Ben Hamed, S. (2022). Prefrontal control of proactive and reactive mechanisms of visual suppression. *Cerebral Cortex*, *32*(13), 2745–2761. 10.1093/cercor/bhab378 10.1093/cercor/bhab378 PMC9247412 34734977

[CR16] Ferrante, O., Patacca, A., Di Caro, V., Della Libera, C., Santandrea, E., & Chelazzi, L. (2018). Altering spatial priority maps via statistical learning of target selection and distractor filtering. *Cortex,**102*, 67–95. 10.1016/j.cortex.2017.09.027 29096874 10.1016/j.cortex.2017.09.027

[CR17] Ferrari, V., Codispoti, M., & Bradley, M. M. (2017). Repetition and ERPs during emotional scene processing: A selective review. *International Journal of Psychophysiology,**111*, 170–177. 10.1016/j.ijpsycho.2016.07.496 27418540 10.1016/j.ijpsycho.2016.07.496

[CR18] Gao, Y., & Theeuwes, J. (2020). Independent effects of statistical learning and top-down attention. *Attention, Perception, & Psychophysics,**82*(8), 3895–3906. 10.3758/s13414-020-02115-x 10.3758/s13414-020-02115-x PMC7593392 32909086

[CR19] Geng, J. J. (2014). Attentional mechanisms of distractor suppression. *Current Directions in Psychological Science,**23*(2), 147–153. 10.1177/0963721414525780

[CR20] Geng, J., Won, B.-Y., & Carlisle, N. (2019). Distractor ignoring: Strategies, learning, and passive filtering. *Current Directions in Psychological Science,**28*(6), 600–606. 10.1177/0963721419867099 33758472 10.1177/0963721419867099 PMC7983343

[CR21] Goldfarb, L., & Henik, A. (2013). The effect of a preceding cue on the conflict solving mechanism. *Experimental Psychology,**60*(5), 347–353. 10.1027/1618-3169/a000205 23628698 10.1027/1618-3169/a000205

[CR22] Grimshaw, G. M., Devue, C., Kranz, L. S., Jenkins, D. C., O’Connell, A., & Carmel, D. (2020). Cognitive control of emotional distractors in central vision [Manuscript in preparation]. School of Psychology, Te Herenga Waka Victoria University of Wellington.

[CR23] Grimshaw, G. M., Kranz, L. S., Carmel, D., Moody, R. E., & Devue, C. (2018). Contrasting reactive and proactive control of emotional distraction. *Emotion,**18*(1), 26–38. 10.1037/emo0000337 28604035 10.1037/emo0000337

[CR24] Huffman, G., Rajsic, J., & Pratt, J. (2019). Ironic capture: Top-down expectations exacerbate distraction in visual search. *Psychological Research Psychologische Forschung,**83*(5), 1070–1082. 28916853 10.1007/s00426-017-0917-z

[CR25] Kim, H., Ogden, A., & Anderson, B. A. (2023). Statistical learning of distractor shape modulates attentional capture. *Vision Research,**202*, Article 108155. 10.1016/j.visres.2022.108155 36417810 10.1016/j.visres.2022.108155 PMC9791481

[CR26] Kim, A., & Anderson, B. (2022). Systemic effects of selection history on learned ignoring. *Psychonomic Bulletin & Review,**29*(4), 1347–1354. 10.3758/s13423-021-02050-4 35112310 10.3758/s13423-021-02050-4 PMC9343477

[CR27] Kleinsorge, T. (2007). Anticipatory modulation of interference induced by unpleasant pictures. *Cognition and Emotion,**21*(2), 404–421. 10.1080/02699930600625032

[CR28] Kleinsorge, T. (2009). Anticipation selectively enhances interference exerted by pictures of negative valence. *Experimental Psychology,**56*(4), 228–235. 10.1027/1618-3169.56.4.228 19439394 10.1027/1618-3169.56.4.228

[CR29] Lahav, A., Makovski, T., & Tsal, Y. (2012). White bear everywhere: Exploring the boundaries of the attentional white bear phenomenon. *Attention, Perception, & Psychophysics,**74*(4), 661–673. 10.3758/s13414-012-0275-2 10.3758/s13414-012-0275-2 22323061

[CR30] Lang, P. J., Bradley, M. M., & Cuthbert, B. N. (2008). International Affective Picture System (IAPS): Affective ratings of pictures and instruction manual. *University of Florida, Gainesville. FL, Technical Report A-8.*

[CR31] Locke, H. S., & Braver, T. S. (2008). Motivational influences on cognitive control: Behavior, brain activation, and individual differences. *Cognitive, Affective, & Behavioral Neuroscience,**8*(1), 99–112. 10.3758/CABN.8.1.99 10.3758/cabn.8.1.99 18405050

[CR32] Makovski, T. (2019). Preparing for distraction: Attention is enhanced prior to the presentation of distractors. *Journal of Experimental Psychology: General,**148*(2), 221–236. 10.1037/xge0000509 30346200 10.1037/xge0000509

[CR33] Makovski, T., & Chajut, E. (2021). Preparing for the worst: Attention is enhanced prior to any upcoming emotional or neutral stimulus. *Psychological Science,**32*(2), 256–266. 10.1177/0956797620963612 33400635 10.1177/0956797620963612 PMC7882998

[CR34] Marini, F., van den Berg, B., & Woldorff, M. G. (2015). Reward prospect interacts with trial-by-trial preparation for potential distraction. *Visual Cognition,**23*(1–2), 313–335. 10.1080/13506285.2015.1023387 26180506 10.1080/13506285.2015.1023387 PMC4500291

[CR35] Micucci, A., Ferrari, V., De Cesarei, A., & Codispoti, M. (2020). Contextual modulation of emotional distraction: Attentional capture and motivational significance. *Journal of Cognitive Neuroscience,**32*(4), 621–633. 10.1162/jocn_a_01505 31765599 10.1162/jocn_a_01505

[CR36] Moher, J., & Egeth, H. E. (2012). The ignoring paradox: Cueing distractor features leads first to selection, then to inhibition of to-be-ignored items. *Attention, Perception, & Psychophysics,**74*(8), 1590–1605. 10.3758/s13414-012-0358-0 10.3758/s13414-012-0358-0 22893004

[CR37] Morey, R. D. (2008). Confidence intervals from normalized data: A correction to Cousineau (2005). *Tutorials in Quantitative Methods for Psychology*, *4*(2), 61–64. 10.20982/tqmp.04.2.p061

[CR38] Murphy, J., Devue, C., Corballis, P. M., & Grimshaw, G. M. (2020). Proactive control of emotional distraction: Evidence from EEG alpha suppression. *Frontiers in Human Neuroscience,**14*, 318. 10.3389/fnhum.2020.00318 33013338 10.3389/fnhum.2020.00318 PMC7461792

[CR39] Padmala, S., Sirbu, M., & Pessoa, L. (2017). Potential reward reduces the adverse impact of negative distractor stimuli. *Social Cognitive and Affective Neuroscience,**12*(9), 1402–1413. 10.1093/scan/nsx067 28505380 10.1093/scan/nsx067 PMC5629819

[CR40] Peirce, J., Gray, J. R., Simpson, S., MacAskill, M., Höchenberger, R., Sogo, H., Kastman, E., & Lindeløv, J. K. (2019). PsychoPy2: Experiments in behavior made easy. *Behavior Research Methods,**51*(1), 195–203. 10.3758/s13428-018-01193-y 30734206 10.3758/s13428-018-01193-y PMC6420413

[CR41] Pool, E., Brosch, T., Delplanque, S., & Sander, D. (2016). Attentional bias for positive emotional stimuli: A meta-analytic investigation. *Psychological Bulletin,**142*(1), 79–106. 10.1037/bul0000026 26390266 10.1037/bul0000026

[CR42] R Core Team (2022). R: A language and environment for statistical computing. R Foundation for Statistical Computing, Vienna, Austria. URL https://www.R-project.org/.

[CR43] Schmidts, C., Foerster, A., Kleinsorge, T., & Kunde, W. (2020). Proactive control of affective distraction: Experience-based but not expectancy-based. *Cognition,**194*, Article 104072. 10.1016/j.cognition.2019.104072 31520864 10.1016/j.cognition.2019.104072

[CR44] Stilwell, B. T., Bahle, B., & Vecera, S. P. (2019). Feature-based statistical regularities of distractors modulate attentional capture. *Journal of Experimental Psychology: Human Perception and Performance,**45*(3), 419–433. 10.1037/xhp0000613 30802131 10.1037/xhp0000613

[CR45] The jamovi project (2024). *jamovi* (Version 2.5) [Computer Software]. Retrieved from https://www.jamovi.org

[CR46] Tsal, Y., & Makovski, T. (2006). The attentional white bear phenomenon: The mandatory allocation of attention to expected distractor locations. *Journal of Experimental Psychology: Human Perception and Performance*, *32*(2), 351–363. Scopus. 10.1037/0096-1523.32.2.351 10.1037/0096-1523.32.2.351 16634675

[CR47] Vatterott, D. B., & Vecera, S. P. (2012). Experience-dependent attentional tuning of distractor rejection. *Psychonomic Bulletin & Review,**19*(5), 871–878. 10.3758/s13423-012-0280-4 22696250 10.3758/s13423-012-0280-4

[CR48] Walsh, A. T., Carmel, D., Harper, D., & Grimshaw, G. M. (2018). Motivation enhances control of positive and negative emotional distractions. *Psychonomic Bulletin & Review,**25*(4), 1556–1562. 10.3758/s13423-017-1414-5 29299776 10.3758/s13423-017-1414-5

[CR49] Walsh, A. T., Carmel, D., & Grimshaw, G. M. (2019). Reward elicits cognitive control over emotional distraction: Evidence from pupillometry. *Cognitive, Affective, & Behavioral Neuroscience,**19*, 537–554. 10.3758/s13415-018-00669-w 10.3758/s13415-018-00669-w 30488225

[CR50] Walsh, A. T., Carmel, D., Harper, D., Bolitho, P., & Grimshaw, G. M. (2021). Monetary and non-monetary rewards reduce attentional capture by emotional distractors. *Cognition and Emotion,**35*(1), 1–14. 10.1080/02699931.2020.1802232 32762297 10.1080/02699931.2020.1802232

[CR51] Wang, B., & Theeuwes, J. (2018a). How to inhibit a distractor location? Statistical learning versus active, top-down suppression. *Attention, Perception, & Psychophysics,**80*(4), 860–870. 10.3758/s13414-018-1493-z 10.3758/s13414-018-1493-z 29476331

[CR52] Wang, B., & Theeuwes, J. (2018b). Statistical regularities modulate attentional capture. *Journal of Experimental Psychology: Human Perception and Performance,**44*(1), 13–17. 10.1037/xhp0000472 29309194 10.1037/xhp0000472

[CR53] Willenbockel, V., Sadr, J., Fiset, D., Horne, G. O., Gosselin, F., & Tanaka, J. W. (2010). Controlling low-level image properties: The SHINE toolbox. *Behavior Research Methods,**42*(3), 671–684. 10.3758/BRM.42.3.671 20805589 10.3758/BRM.42.3.671

[CR54] Won, B.Y., Kosoyan, M., & Geng, J. J. (2019). Evidence for second-order singleton suppression based on probabilistic expectations. *Journal of Experimental Psychology. Human Perception and Performance*, *45*(1), 125–138. 10.1037/xhp0000594 10.1037/xhp0000594 PMC8815295 30596437

