# Supplementary material for: Expectations don’t protect us from emotional distractions
Source: Atten Percept Psychophys. 2025 May 23;87(5):1544–60. doi: 10.3758/s13414-025-03085-8 (PMC12204873; doi:10.3758/s13414-025-03085-8)
Supplement: Supplementary file 1 — Supplementary file1 (DOCX 15 KB) [file 13414_2025_3085_MOESM1_ESM.docx]

**Supplementary Material**

**Table S1**
*Mean (SD) IAPS image ratings for valence and arousal, (derived from Lang et al., 2008)*

|  | Valence | Arousal |
| --- | --- | --- |
| Image set | Mean Valence (SD) | Mean Arousal (SD) |
| Female |  |  |
| Neutral | 5.01 (0.34) | 3.07 (0.32) |
| Negative | 1.64 (0.21) | 6.53 (0.42) |
| Baseline | 5.21 (0.32) | 3.36 (0.54) |
| Male |  |  |
| Neutral | 4.97 (0.38) | 3.16 (0.42) |
| Negative | 1.83 (0.18) | 6.43 (0.41) |
| Baseline | 5.31 (0.39) | 3.51 (0.31) |

*Note.* IAPs image sets used; Female Neutral = (2026, 2102, 2221, 2305, 2393, 2397, 2411, 2512, 2593, 2595, 2745.1, 2840), Female Negative = (3015, 3030, 3059, 3103, 3131, 3140, 3150, 3195, 3550.1, 9253, 9405, 9420), Female Baseline = (2107, 2191, 2200, 2214, 2235, 2273, 2377, 2441, 2506, 2870, 7493, 8312), Male Neutral = (2026, 2102, 2104, 2221, 2393, 2397, 2411, 2512, 2593, 2595, 2745.1, 2840), Male Negative = (3000, 3015, 3053, 3060, 3069, 3071, 3080, 3100, 3120, 3130, 3131, 9410), Male Baseline = (2107, 2191, 2200, 2214, 2235, 2273, 2377, 2441, 2506, 2870, 7493, 8312). Baseline sets were implemented in Experiment 3 baseline trials only.

**Table S2**
*Responses to Question 5 of the post-experiment questionnaires, separated by response similarity*

|  | Predictable low-frequency | Unpredictable high-frequency | Unpredictable low-frequency | Total |
| --- | --- | --- | --- | --- |
| Response regarding distractors | 4 | 2 | 7 | 13 |
| Response regarding the task relevant stimuli | 1 | 1 | 0 | 2 |
| Response regarding conscious preparation for upcoming trials | 3 | 0 | 0 | 3 |
| Total | 8 | 3 | 7 | 18 |
